# Associations between measures of socio-economic position and sustainable dietary patterns in the NutriNet-Santé study

**DOI:** 10.1017/S1368980022002208

**Published:** 2023-05

**Authors:** Julia Baudry, Benjamin Allès, Brigitte Langevin, Anouk Reuzé, Joséphine Brunin, Mathilde Touvier, Serge Hercberg, Denis Lairon, Sandrine Péneau, Philippe Pointereau, Emmanuelle Kesse-Guyot

**Affiliations:** 1 Sorbonne Paris Nord University, Inserm U1153, INRAE U1125, Cnam, Nutritional Epidemiology Research Team (EREN), Epidemiology and Statistics Research Centre, Université Paris Cité (CRESS), UFR SMBH 74, Rue Marcel Cachin, Bobigny 93017, France; 2 Solagro, Toulouse, France; 3 ADEME (Agence de l’Environnement et de la Maîtrise de l’Energie), Angers, France; 4 Public Health Department, Avicenne Hospital, AP-HP, Bobigny, France; 5 Aix Marseille University, Inserm, INRAE, C2VN, Marseille, France

**Keywords:** Diet sustainability, Socio-economic status, Diet quality, Environmental sustainability, Observational study

## Abstract

**Objective:**

We aimed to explore the relationship between socio-economic characteristics and sustainable dietary patterns.

**Design:**

Dietary data were derived from a web-based FFQ. Diet sustainability was evaluated using a modified Sustainable Diet Index, comprising nutritional, environmental and cultural components (higher scores expressing higher sustainability). The socio-economic position markers were education, household income and occupation status. Multi-adjusted linear and Poisson regression models were used to assess the cross-sectional association of the markers of socio-economic status with a sustainable diet and sustainability subcomponents, respectively.

**Setting:**

France.

**Participants:**

29 119 NutriNet-Santé participants.

**Results:**

Individuals with a more sustainable diet had slightly higher diet monetary cost, lower total energy intake and consumed less animal-based foods than their counterparts. Lower education level was associated with lower overall diet sustainability (*β*_primary *v*. postgraduate_ = -0·62, 95 % CI (-0·72, −0·51)) and nutrition, socio-cultural and environmental subscores. Manual workers and employees had a lower modified Sustainable Diet Index than intermediate professionals (*β*_manual workers *v*. intermediate professionals_ = -0·43, 95 % CI (−0·52, −0·33) and *β*_employees *v*. intermediate professionals_ = -0·56, 95 % CI (−0·64, −0·48)). Participants with the lowest *v*. highest incomes had a higher environmental subscore but a lower socio-cultural subscore, whereas the results were less marked for occupational status.

**Conclusions:**

Overall, our results documented associations between socio-economic status and the level of diet sustainability, arguing for the implementation of appropriate food policies to promote sustainable diets at lower cost.

Health and diet inequalities are closely related to socio-economic status^(1)^. In Westernised countries, greater prevalence of non-communicable diseases has been repeatedly observed among socio-economically disadvantaged populations, with nutritional factors being important risk factors^(2)^. Studies investigating the links between dietary factors and socio-economic groups are plentiful among children^(3)^, but more sparse among adults. These have generally focused on dietary quality, by concentrating either on the intake of unhealthy or healthy food groups and nutrients^(4,5)^ or on dietary patterns, using e.g. *a posteriori* defined healthy dietary patterns or *a priori* scores reflecting adherence to official dietary recommendations^(6,7)^.

However, over the past decade, numerous studies have underlined the environmental damage caused by our current food systems, urging for a transition to healthy and environmentally sustainable dietary patterns^(8–10)^. In that context, the definition of sustainable diets stated by the FAO goes beyond the health and nutritional values of diets and also encompasses environmental, socio-economic and cultural components^(8)^. Many studies have showed co-benefits of plant-based dietary patterns on planetary and human health^(9–12)^, since plant-based diets are generally both healthy and have fewer impacts on the environment than animal-based diets. On the other hand, other studies point out potential conflicts between the dimensions of food sustainability; the different dimensions do not necessarily align with each other^(13–15)^. For instance, in a study by Clark *et al.* ^(13)^, sugar-sweetened beverages had among the lowest environmental impacts for the studied indicators despite being associated with higher risk of diseases, and the opposite was true for fish.

While lower education status linked to unhealthy diets, diets of disadvantaged individuals might not be systematically more impactful on the environment. For instance, in the Third French Individual and National Food Consumption (INCA 3) survey (2014–2015), French individuals with higher education level consumed more fruits and vegetables (which have generally low environmental footprints), but also more cheese, dairy products (which have high environmental footprint) or chocolate. In contrast, individuals with a lower educational level consumed more products with low environmental impacts such as soda and potatoes but also ate more high-impact products such as meat (excluding poultry)^(16)^.

Different factors measuring socio-economic position (i.e. occupational position, household income or education attainment) may be differentially associated with sustainable diets, as they affect food choices in different ways^(17)^. More highly educated individuals are generally more aware of the recommended diets^(18)^ as well as potentially of the impacts of food on the environment. They generally exhibit higher food and health literacy^(17)^, i.e. the ‘capacity to obtain, process and understand basic health information and services needed to make appropriate health decisions’.

Household income influences a person’s ability to afford a healthy and possibly a more sustainable diet. For example, the price of organic products, with their potential health and environmental benefits^(19)^, is often seen as a barrier for low-income households. Occupation type could also play a role in food intakes depending on the culture and the environment of the workplace. Yet limited evidence is available on the associations between overall diet sustainability, using composite indexes and socio-economic status^(20)^.

Hence, the present study aimed to examine how socio-economic status, measured as income, education and occupational status, relates to sustainable dietary patterns, using an index accounting for various dimensions of diet sustainability, in a large cohort of French adults. We first investigated the association of socio-economic status with overall diet sustainability. A second objective was to more specifically examine the relation between socio-economic position and the different components of diet sustainability.

## Methods

### Study population

We used data from the NutriNet-Santé study. The NutriNet-Santé study is an ongoing web-based prospective cohort study launched in 2009 in France, established to investigate the associations between nutrition and health as well as determinants of dietary behaviours. Participants are Internet-using adult volunteers recruited from the general population. At recruitment and during follow-up, lifestyle, medical history and socio-economic factors are collected via online questionnaires available on a web platform (https://etude-nutrinet-sante.fr/). Additional information is also collected regarding dietary practices and behavioural issues related to nutrition and health. The study protocol and the procedures have been fully described previously^(21)^.

### Assessment of socio-demographic variables and socio-economic position

Socio-demographic characteristics, including sex, age, marital status, presence of children in the household and residential area, were assessed using self-administered questionnaires^(22)^.

Socio-economic position included monthly income per household unit, highest educational attainment and current or last (in case of retirement or unemployment) occupational category. More specifically, in the self-administered questionnaires, participants completed their total income per month assessed from various sources (salary, rental income, family allowance or social benefits). Monthly household income was then defined by household unit, according to the definition of the National Institute of Statistics and Economic Studies (INSEE)^(23)^, i.e. 1 household unit was allocated to the first adult in the household, 0·5 to other individuals aged ≥14 years and 0·3 to children aged below 14 years. Household income was classified into five groups (<1200 €/month; 1200–1800 €/month; 1800–2700 €/month; >2700 €/month and refuse to declare). Education level was categorised as primary level education, secondary level education, undergraduate (up to 3 years after high school diploma) and postgraduate (>3 years after high-school diploma). We created a six-category variable for occupational status based on INSEE categories’ definition: self-employed (craftsman, shopkeeper, entrepreneur and farmer); managerial staff/intellectual profession; intermediate profession; employee; manual worker and never-employed (homemaker, student and disabled)^(23)^.

Individual data were extracted from the questionnaire completed on the date closest to the date of completion of the FFQ described below.

### Organic FFQ

Dietary information was collected using a 264-item self-administered FFQ (Org-FFQ) designed to evaluate individual’s habitual diet over the preceding year. The Org-FFQ has been described elsewhere^(24)^. In brief, the Org-FFQ was developed within the frame of the BioNutriNet project^(19)^ and administered in 2014. It is based on an existing previously validated semi-quantitative FFQ^(25)^. Additional questions regarding organic food consumption frequency were included. More specifically, for each item, participants were asked to provide the quantity consumed and the frequency of consumption (per day, week, month or per year). Quantity of food consumed was estimated using standard portion sizes (e.g. a slice of bread) or coloured photographs displayed to estimate the quantity consumed among seven portion sizes. Participants were also asked to provide their organic food intake frequency for each food and beverage item available as organic option, through a 5-point scale, ranging from ‘never’ to ‘always’ (intermediate modalities included ‘rarely, ‘half-of-the-time’ and ‘often’). Total food intake was obtained by multiplying the frequency of consumption by the amount of food consumed. Organic food consumption for each item was estimated by assigning the following proportions 0, 0·25, 0·5, 0·75 and 1 to the corresponding frequency modalities. The share of organic food in the diet was then obtained by dividing the total organic food intake (g/d) out of the total intake excluding water (g/d).

### The sustainable diet index

We used the Sustainable Diet Index (SDI) whose construction and validation have been extensively described elsewhere^(26)^. In short, the original SDI includes seven indicators categorised into four equally weighted subscores, based on the FAO’s definition of sustainable diets^(8)^ which encompasses four pillars (nutrition, environment, economy and socio-cultural aspects).

The nutritional component is composed of an indicator reflecting the gap between energy needs (estimated using Schofield equation^(27)^) and energy intake, and of the PANDiet (Diet Quality Index Based on the Probability of Adequate Nutrient Intake) index, which reflects adherence to French nutritional references^(28)^. The environmental component includes the pReCiPe (partial ReCiPe)^(29)^ (a synthetic environmental score, including greenhouse gas emissions (GHGe), cumulative energy demand and land occupation, weighted by means of coefficients) and organic food consumption as a proxy for greater biodiversity^(30)^. The socio-cultural component includes an indicator related to the type of food supply and a score related to consumption frequency of ready-made foods. In the original SDI, the economic component is composed of an indicator defined as the proportion of income devoted to food. In the present work, we elected to not include affordability (i.e. proportion of the income devoted to diet) since socio-economic markers would be highly correlated to it. Therefore, we computed a modified Sustainable Diet Index (mSDI), by removing the economic component.

A detailed description pertaining to the assessment of the different indicators included in the original SDI is given in the Supplemental material.

For indicators presumed to be ‘favourable’ for sustainability, 1 point was assigned to participants in the lowest quintile, 2 points to those in the second quintile and so on. The scoring was inverted for indicators considered as ‘detrimental’. The seven indicators were weighted so that each component had the same weight and had a range comprised between 1 and 5. The final score was obtained by summing up the points for each subscore and ranged from 4 (low sustainability) to 20 (high sustainability). Quintile values retrieved during the development of the score were used^(26)^. The original scoring is presented in Supplemental Table 1.

The modified SDI ranged (without the affordability component) from 3 to 15.

### Selection of the study sample and weighting procedure

Of the 37 685 NutriNet-Santé participants who responded to the Org-FFQ from June to December 2014, 29 119 were included in the analyses. Individuals with missing covariates, those with an implausible ratio of energy intake to energy requirement estimated by Schofield equations^(27)^ (using predefined cut-offs^(24)^), those not living in mainland France, those with unavailable data on food source supply and for the computation of the SDI were excluded from the analysis. Additionally, we also excluded unemployed and retired participants whose last occupational status could not be identified (see online Supplemental Fig. 1).

We applied a weighting procedure to all analyses in order to better fit the proportions of socio-demographic categories existing in the French population. To do so, using an iterative proportional fitting procedure, a weighting was performed separately for each sex, according to age, occupational status, education level, residential area, presence of children in the household (<18 years) and marital status, attributing a weight to each individual (SAS macro %CALMAR and the 2009 national census INSEE data).

### Statistical analysis

General characteristics of the study participants are presented as mean (sd) or percentage according to quintiles of the mSDI.

Collinearity between the three socio-economic indicators was examined using the variance inflation factor. Variance inflation factor was below 4 in all regression models, suggesting no substantial collinearity and the three indicators were included simultaneously in the models.

Q–Q plots and histograms were used to visualise whether the residuals were normally distributed for all unweighted regression models conducted.

Associations of general characteristics across weighted mSDI quintiles were tested with linear contrast tests for continuous variables, Mantel–Haenszel chi-square trend tests or chi-square tests for ordinal and categorical variables, respectively.

Mean contribution of each food group to total consumption (g/d), cost (€/d) and energy intake (kcal/d) across weighted quintiles was calculated. Average diet monetary cost (€/d) distinguishing the farming system of food consumed, according to mSDI quintiles, was also computed.

For the main analysis, using the mSDI as dependent variable, normality assumptions were fulfilled and therefore linear models were fitted to estimate the effect estimates (*β*) and 95 % CI for the association of the three individual socio-economic indicators and the mSDI (modelled as a continuous variable), in the full sample and by sex. Models were adjusted for (sex), age, total energy intake, parental status, residential area and the two remaining socio-economic factors.

Due to their distribution, the individual subscores (namely environment, nutritional and socio-cultural subscores) were treated as ‘count-like’ discrete variables and Poisson regressions were constructed to assess the associations between socio-economic indicators and individual subscores. In doing so, we sought to assess whether any of the socio-economic factors were most strongly related to any of the individual subscores.

Statistical significance was set at *P*−value < 0·05. Data treatment and statistical analyses were performed using SAS (version 9.4, SAS Institute Inc.) and R (version 3.6.2).

## Results

### Sample description

Table 1 displays the characteristics of the study sample across weighted mSDI quintiles. Individuals in Q5 (with a more sustainable diet), compared to individuals in Q1, were more often females, aged 65 years or older and less likely to have a child at home. No association was found between residential area and the mSDI. The proportion of individuals with primary education level was highest in Q2 while the proportion of postgraduate participants increased with increasing mSDI. The highest percentage of low-income individuals was found in Q2 whereas participants unwilling to provide their income were the most numerous in Q5. Q1 and Q2 showed the highest proportion of manual workers and Q5 the highest of intermediate profession.

**Table 1:** Characteristics of participants according to mSDI quintiles (weighted data)*

	Q1 3–6·25		Q2 6·25–7·5		Q3 7·5–8·75		Q4 8·75–10·5		Q5 10·5–15	
	Mean	sd	Mean	sd	Mean	sd	Mean	sd	Mean	sd
mSDI	5·20	1·09	6·94	0·45	8·18	0·38	9·60	0·45	11·91	0·89
Environment subscore	1·55	0·75	2·06	0·93	2·72	0·91	3·24	0·78	4·23	0·61
Socio-cultural subscore	1·89	0·92	2·64	1·12	2·81	1·01	3·23	0·87	3·93	0·76
Nutritional subscore	1·76	0·85	2·24	0·96	2·65	0·88	3·13	0·75	3·74	0·69
	%		%		%		%		%	
Female	36·57		39·93		52·41		60·95		71·63	
Age categories										
18–30 years	17·12		15·37		15·61		12·88		17·29	
30–50 years	45·57		42·36		32·34		32·05		28·72	
50–65 years	23·32		24·45		29·99		26·69		28·57	
≥65 years	13·99		17·81		22·05		28·38		25·42	
At least one child at home (yes)	46·14		47·85		36·27		27·62		25·24	
Residential area										
Rural community	25·31		23·74		25·83		25·92		26·23	
Urban unit with less than 20 000 inhabitants	18·53		15·43		13·90		16·80		14·58	
Urban unit with 20 000 to 200 000 inhabitants	16·24		17·29		16·84		18·60		16·50	
Urban unit with more than 200 000 inhabitants	39·91		43·54		43·43		38·68		42·69	
Educational level										
Primary	25·96		31·16		27·02		23·71		30·55	
Secondary	55·06		47·08		46·29		48·50		40·29	
Undergraduate	9·15		11·18		13·66		13·18		12·13	
Postgraduate	9·83		10·58		13·03		14·61		17·02	
Income per household unit										
<1200 €/month	27·16		33·79		22·51		21·35		21·73	
1200–1800 €/month	30·34		29·51		28·58		26·16		26·46	
1800–2700 €/month	23·90		19·81		25·81		24·88		27·34	
>2700 €/month	10·38		12·09		16·68		19·60		16·16	
Refuse to declare	8·22		4·79		6·42		8·00		8·31	
Occupational status										
Self-employed (e.g. farmer/entrepreneur)	5·48		6·48		6·19		6·16		9·87	
Never-employed (e.g. student)	9·09		9·30		10·40		8·21		10·55	
Manual worker	23·80		25·23		12·35		9·93		8·91	
Employee/blue collar	29·94		27·31		30·29		32·91		27·45	
Intermediate profession	18·29		18·33		24·70		23·38		25·07	
Managerial staff/intellectual profession	13·40		13·35		16·06		19·40		18·15	

mSDI, modified sustainable diet index.

* Values are means (sd) or percent, as appropriate. All *P*-values < 0·0001, except residential area (*P*-value = 0·84). *P*-values refer to *P*-trend estimated using linear contrast tests for continuous variables, Mantel–Haenszel chi-square trend tests and chi-square tests for ordinal and categorical variables, respectively.

### Diet-related characteristics across mSDI quintiles

**Fig. 1:**
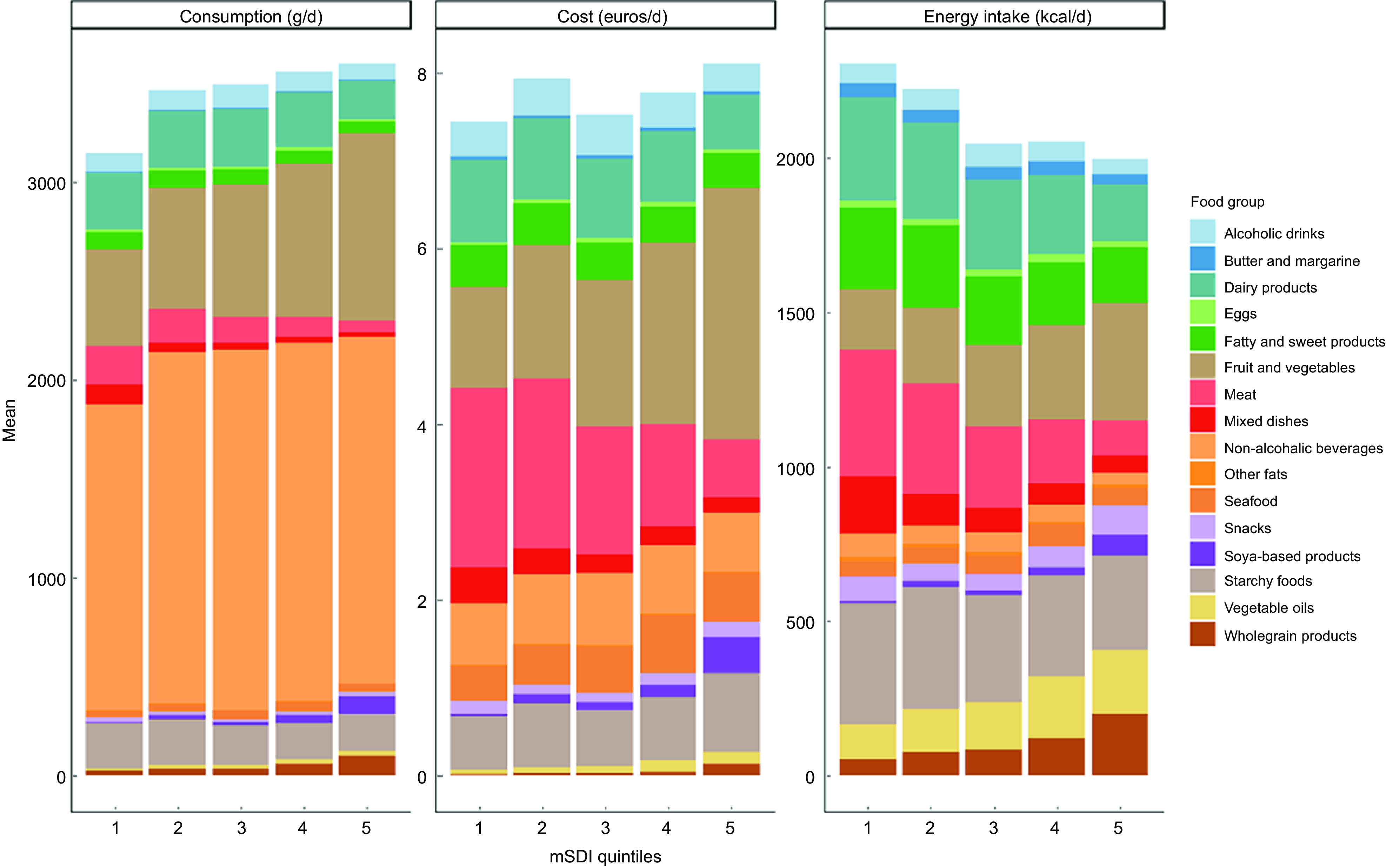
Average food intake (g/d), diet monetary cost (€/d) and energy intake (kcal/d), according to mSDI quintiles (weighted data)* Abbreviation: mSDI, modified Sustainable Diet Index. All *P*-values for linear contrast < 0·0001. *Data are unadjusted.

Daily food intake, diet monetary cost and energy intake, according to mSDI quintiles are shown in Fig. 1. Total food intake ranged from 3151 (1639) g/d (Q1) to 3600 (844) g/d (Q5). Diet monetary cost ranged from 7·43 (4·14) €/d in Q1 to 8·10 (2·38) €/d in Q5. Meat was the most important contributor to total diet monetary cost in Q1, whereas intake of fruit and vegetables was the most important contributor in Q5. Total energy intake decreased along with higher mSDI. Participants in Q5 showed a higher contribution to energy intake from fruit and vegetables than those in Q1 (approximately 20 % for Q5 *v*. < 10 % for Q1) and a threefold lower contribution to energy intake from meat. Unsurprisingly, diet monetary cost dedicated to organic food increased with increasing mSDI (see online Supplemental Fig. 2).

### Associations between socio-economic factors and mSDI

**Fig. 2:**
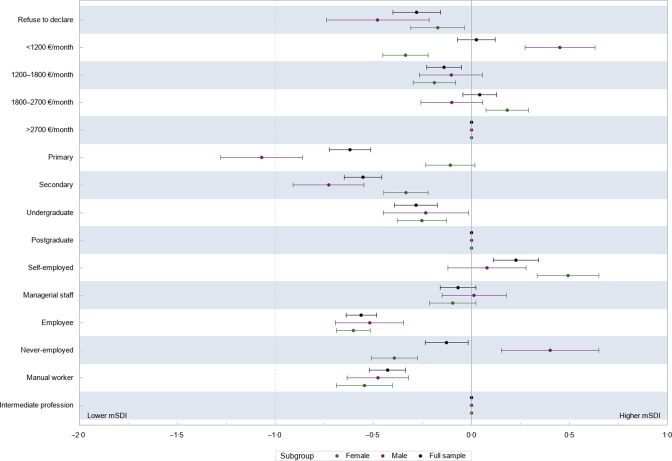
Association between socio-economic factors and mSDI, linear regression coefficients and 95 % CI (weighted data)* Abbreviation: mSDI, modified Sustainable Diet Index. *Multivariable linear regression for the association between the mSDI and each socio-economic factor. Multivariable-adjusted models include (sex), age, total energy intake, parental status, residential area and socio-economic factors. The socio-economic factors are mutually adjusted for the remaining factors.

Figure 2 presents the association between the three measures of socio-economic factors and the mSDI. Individuals with monthly income comprised between 1800 and 2700 € and those with an income below <1200 € did not have significantly different mSDI compared to high-income individuals (>2700 €/month) (whole sample). The mSDI for individuals with intermediate income (1200–1800 €/month) was lower than for high-income individuals. Some differences were observed across sex, in particular, low-income males showed mSDI higher than the reference (high-income individuals) while the opposite held for females. Regarding education, lower educational level was overall associated with lower mSDI (whole sample and both sexes). Of note, no difference was detected when comparing low-educated to postgraduate females. Employees and manual workers (full sample, males and females) had a lower mSDI score than intermediate professionals. Females who never worked had a mSDI score lower than intermediate professionals whereas the opposite was observed for males. Compared to the reference, managerial staff did not have different mSDI scores.

The environmental subscore was higher for individuals with income below 2700 €/month than for the reference category (>2700 €/month) while it was lower for individuals with low-educational levels compared to postgraduate individuals (Table 2). Employees, manual workers and managerial staff had an environment subscore that was lower than intermediate professionals. Individuals with low-income or low-education level had lower socio-cultural subscore compared to those with high income or postgraduate. Never-employed, manual workers and employees had a lower socio-cultural subscore than intermediate professionals while this subscore was higher for self-employed individuals. Only individuals with intermediate income (1800–2700 €/month) had a significant lower nutritional subscore compared to high-income levels. Individuals with low educational level (primary and secondary) had lower nutritional subscore in comparison to postgraduate participants. Self-employed and employees had a lower nutrition subscore than participants belonging to the intermediate profession category while no significant associations were observed for other occupational categories. Supplemental Table 2 shows the relationships between socio-economic factors and individual subscores according to sex.

**Table 2:** Association between socio-economic factors and individual subscores, Poisson regression coefficients and 95 % CI (weighted data)*

	Environment subscore		Socio-cultural subscore		Nutritional subscore	
	β	95 % CI	β	95 % CI	β	95 % CI
Income per household unit						
<1200 €/month	0·0742	0·0484, 0·1001	−0·0619	−0·087, −0·037	0·0002	−0·026, 0·0261
1200–1800 €/month	0·0401	0·0164, 0·0638	−0·0799	−0·103, −0·057	−0·0038	−0·027, 0·0199
1800–2700 €/month	0·0471	0·0237, 0·0704	0	−0·022, 0·0220	−0·0318	−0·055, −0·008
>2700 €/month	Ref		Ref		Ref	
Refuse to declare	−0·0196	−0·053, 0·0135	−0·0569	−0·089, −0·025	−0·0193	−0·052, 0·0136
Educational level						
Primary	−0·0532	−0·082, −0·025	−0·0905	−0·118, −0·063	−0·0706	−0·099, −0·042
Secondary	−0·0595	−0·085, −0·034	−0·0837	−0·108, −0·059	−0·0521	−0·078, −0·026
Undergraduate	−0·0334	−0·062, −0·005	−0·0447	−0·073, −0·017	−0·0196	−0·049, 0·0099
Postgraduate	Ref		Ref		Ref	
Occupational status						
Self-employed (e.g. farmer/entrepreneur)	0·0045	−0·026, 0·0352	0·0956	0·0668, 0·1244	−0·0327	−0·065, −0·001
Never-employed (e.g. student)	−0·0249	−0·054, 0·0044	−0·0377	−0·067, −0·009	0·0206	−0·009, 0·0501
Manual worker	−0·0986	−0·124, −0·073	−0·0434	−0·068, −0·019	−0·0239	−0·050, 0·0021
Employee/blue collar	−0·0672	−0·088, −0·047	−0·0947	−0·115, −0·074	−0·0354	−0·056, −0·015
Intermediate profession	Ref		Ref		Ref	
Managerial staff/intellectual profession	−0·0263	−0·051, −0·002	−0·0135	−0·037, 0·0100	0·0196	−0·005, 0·0443

* Multivariable Poisson regression for the association between the individual component and each socio-economic factor. Multivariable-adjusted models include sex, age, total energy intake, parental status, residential area and socio-economic factors. The socio-economic factors are mutually adjusted for the remaining factors.

Bold denotes significance.

## Discussion

### Summary of the findings

We assessed the relationships between socio-economic status using three indicators and sustainable dietary patterns expressed by the mSDI (the original SDI without the economic dimension). By construction, a more sustainable diet was associated with lower energy intake, lower intakes of animal-sourced foods and higher intakes of fruit and vegetables. A higher mSDI was also linked to a slightly higher monetary diet cost in the crude analysis. After adjusting for multiple factors including total energy intake, we observed a mSDI gradient across educational level. Manual workers and employees had also lower mSDI than intermediate professionals.

### Comparison with studies on overall socio-economic status in relation to diet sustainability as a whole

To our knowledge, studies examining the association between various socio-economic factors and sustainable diets are scarce, and a comparison with those of the literature proves difficult.

However, certain of our findings can be compared in light of socio-economic determinants of dietary patterns since plant-based diets are generally nutritionally and environmentally better^(9–12)^. Socio-economic factors associated with dietary patterns have indeed been extensively described^(1,6)^.

A study has been recently conducted in a representative sample of Iranian households with a comparable research question as ours^(20)^, i.e. examining socio-economic status, using a composite indicator, in relation to overall diet sustainability (assessed based on dietary carbon footprint, dietary water footprint, dietary costs and dietary quality index). Interestingly, the most privileged households had a less sustainable diet, due to their high energy and food intake, especially of animal-based foods, in contrast to our findings. A hypothesis advanced by the authors is that in a middle-income country such as Iran, urbanised and affluent populations might be experiencing the nutrition transition^(31)^ faster than the rest of the population. Apart from the different country context, dietary data were based on household expenses and socio-economic status was assessed as a whole by deriving, through principal component analysis, an aggregated socio-economic index including different socio-economic indicators, limiting the comparison with our work.

### Comparison with studies on education

We observed a graded association between education level and a more sustainable diet. In particular, individuals with low-education had a lower nutritional subscore. While limited data is available regarding sustainable diets, the latter observation is consistent with studies reporting that a higher level of educational attainment is associated with a better adherence to different diet quality indices^(6,7,32)^. Similarly, in the French nationally representative ESTEBAN survey, individuals with higher levels of education reported a diet more in line with the recommendations^(33)^. In particular, higher intake of fruit and vegetables and wholegrain products have been consistently associated with higher socio-economic status^(1,34)^.

Our findings are therefore in line with previous works indicating that individuals with highest education levels are generally better informed about nutritional^(18)^ and health recommendations^(17)^ and more concerned about environmental issues^(35)^, and in turn might be more likely to adopt healthy^(21)^ and pro-environmental eating behaviours. In studies conducted within the NutriNet-Santé cohort, we observed that highly educated organic food consumers were also those the most concerned by ethics and environmental issues and those the most able to recognise official organic labels^(36,37)^.

### Comparison with studies on income

Concerning income, total energy intake, animal-based food demand and a gross domestic product are positively correlated; high-income countries generally displaying a higher consumption of meat and processed food^(38)^. Conversely, in developed nations, within countries, unhealthy diets characterised by energy-dense and nutrient poor foods prevail in low-income category populations^(39)^. Interestingly, in our study, individuals belonging to the lowest income categories did not systematically show a less sustainable diet. It was even the opposite for low-income (<1200 €/month) males in comparison to the highest income group, in spite of the adjustment for age. The strongest negative association with income was nevertheless observed among the potentially vulnerable group of low-income females. Participants with an intermediate income (1200–1800 €/month) had a lower mSDI than their more privileged counterparts. It would appear that it is possible to have a relatively low income and a sustainable diet, indicating that household income should not be too low but not necessarily very high. Of note, our models were multi-adjusted, including for remaining socio-economic factors (i.e. occupational status and education level), which may have attenuated the associations with income. In general, education level appears to be the strongest socio-economic predictor of diet quality^(17)^. For example, in a study conducted in the NutriNet-Santé cohort^(5)^, education level modulated the relationships between income and animal food consumption and appeared to be the strongest predictor of animal food consumption. A study conducted in the United Kingdom based on household purchase data is also worth mentioning^(40)^. The authors reported that sustainable diets (defined as GHGe, cost and land use being below the median and a Diet Quality Index being above the median) were more prevalent in households belonging to the lowest income quintile than the rest of the sample (22 % *v*. 16·6 %).

With regard solely to diet quality in an Australian study, a positive relationship was found between income and diet quality assessed using the Dietary Guideline Index^(32)^ for both males and females while no evident gradient was observed within our study. A possible explanation for this discrepancy between the two studies could be, among other factors, the use of different scores to assess diet quality. We use the PANDiet which is a score reflecting adherence to the French nutrient-based recommendations while the Dietary Guideline Index reflects adherence to food-based recommendations. The general public may be less aware of reference values of vitamins and minerals used for the computation of the PANDiet.

With regard to environment, in our study, low-income individuals had higher scores (i.e. lower environmental impacts) than those in the highest income category. A study conducted in Lima also reported higher income associated with higher GHGe^(41)^. A study conducted in thirty countries indicated that high-income individuals are more concerned about environmental issues^(35)^ than their counterparts. However, in a typology derived from conventional and organic food intake in the NutriNet-Santé study, we observed that individuals with the highest income were not those with the healthiest diets and greater organic food consumption^(36)^.

### Comparison with studies on occupational status

Regarding occupational status belonging to intellectual professions/managerial staff categories was not linked to a more sustainable diet when compared to intermediate professions. Nonetheless, employees, females who have never been employed and manual workers, had a less sustainable diet.

In a study conducted in the NutriNet-Santé cohort^(5)^, in respect of animal food intake, an association was observed between occupation and dairy product consumption. Manual workers ate more cream desserts than managerial staff but were less often yoghurt consumers, suggesting that a disparity may exist in the choice of healthy *v*. unhealthy products rather than on the type of food in the association. In the present work, manual workers and employees had lower diet sustainability, even after adjustment for other socio-economic factors, suggesting a role of occupation beyond merely education and income.

Self-employed (a group which includes varied profiles, including business managers and farmers) had a more sustainable diet than intermediate professions. Since this category gathers highly heterogeneous groups, conclusions can be hardly drawn and any interpretation must be made with cautious.

### Socio-cultural aspects

Individuals with lower socio-economic status had a lower socio-cultural subscore, which included the main food source supply and the consumption frequency of ready-made products.

This seems in accordance with data from the French INCA 3 survey pertaining to behavioural purchase. Indeed, higher level of education (more than 3 years after high school)^(16)^ was related to more frequent purchase in local markets, shops and short commercialisation channels^(16)^.

In a study also conducted within the NutriNet-Santé cohort on >60 000 individuals, belonging to the lowest socio-economic groups (i.e. those belonging to the lowest education, income groups and to the category of female manual and office workers) was associated with a longer time spent on the meal preparation^(42)^, in accordance with another work^(43)^ showing that commitment to meal preparation is generally higher among low-class individuals. However, in the same study^(42)^, it was observed that, compared to managerial staff, female manual and office workers used fewer raw materials or fresh produce to prepare their meals^(42)^, which seems rather consistent with our results.

### Study limitations and strengths

Some limitations of the present work should be stressed. Firstly, dietary data were self-reported and thus subject to measurement errors. In addition, study participants are adult volunteers enrolled in a cohort focusing on nutrition and health and therefore more likely overall to be prone to exhibit heathier dietary habits than the general population^(44)^. The NutriNet-Santé cohort study thus comprises more females and more educated people than the French population. Some population segments, such as highly disadvantaged males are therefore not represented in the cohort. However, to limit these potential biases, we performed a weighting procedure so as to improve the representativeness of the sample compared to the French population. Furthermore, the score was built so that each subcomponent had the same weight, as in the FAO definition. Nonetheless, some subcomponents were composed of very detailed indicators while other indicators were simpler. It would therefore be interesting in the future to include other indicators, such as fair trade, or fine-tune some others, such as the consumption of ready-made products, in order to better take into account the potential conflicts between the different components of sustainability.

A strength of the study is its large sample size, which enabled us to access a wide range and contrasted eating habits and socio-economic profiles and to conduct stratified analyses. In addition, to assess diet sustainability, we used an index based on a validated score^(26)^, which has been associated with long-term health outcomes^(45)^ and encompassing multiple indicators, including three environmental indicators.

### Final considerations

In the present work, educational level appears to be a strong driver of sustainable diets, independent of household income or occupation. Manual workers and employees appeared also to be ‘at-risk’ individuals. In their recent study exploring many drivers of a new dietary transitions for twenty-five countries across the world, Pais *et al*.^(46)^ reported that education along with price changes could promote dietary transition towards a more sustainable and healthy future.

It is important to note that apart from a reduction of animal-based food consumption, not overconsuming is a major lever for reducing diet-related GHGe^(15)^. We observed herein that individuals with a more sustainable diet had a slightly greater diet monetary cost, due most likely to their high proportion of organic food. The cost of organic food was, however, somewhat offset by a lower energy intake and lower intake of animal-based foods, leading to only a slight increase of diet monetary cost. Lower diet quality may be explained in part by food cost^(47)^, high energy-dense food being generally cheaper than healthy products. Cost of healthy and environmentally friendly diets might be higher than unhealthy diets. For instance, higher adherence to food-based dietary guidelines has been associated with greater diet monetary cost^(48)^.

A shift towards sustainable eating patterns is urgent and requires systemic changes, including fiscal policies, appropriate governance as well as public awareness raising^(49)^. The latter alone (information and awareness) might be indeed insufficient, as the transition cannot be left only to the individual, multi-level actions are therefore needed for the transition towards sustainable diets^(49)^. Constraints and representations of sustainable practices of low social class groups may differ from those of ‘dominant’ groups^(50)^, and therefore require targeted strategies. For low social class individuals, sustainable strategies such as, for example seasonal product consumption, waste avoidance or frugal consumption may be preferentially chosen rather than consumption of organic vegetarian products^(50)^. Finally, interventions aiming to foster pro-health or pro-environmental behaviours would need careful attention as their effects can be sometimes divergent (e.g. sugary foods)^(49)^.

## Conclusions

To conclude, while diet, in its nutritional quality dimension, was already a well-documented marker of social inequality, our findings suggest that lower social group individuals, especially manual workers and low educated individuals, have also a less sustainable diet, leading us to advocate for the development and deployment of large nutritional literacy-adapted education programmes combined with appropriate food policy measures, to ensure healthy and environmentally sustainable diets at affordable cost for all. These results go further and illustrate clear associations between socio-economic position and diet sustainability.

## Supplementary material

For supplementary material accompanying this paper visit https://doi.org/10.1017/S1368980022002208. click here to view supplementary material
