# Supplementary material for: Associations between measures of socio-economic position and sustainable dietary patterns in the NutriNet-Santé study
Source: Public Health Nutr. 2022 Oct 10;26(5):965–75. doi: 10.1017/S1368980022002208 (PMC10346073; doi:10.1017/S1368980022002208)
Supplement: Supplementary file 1 [file S1368980022002208sup001.docx]

Associations between measures of socioeconomic position and sustainable dietary patterns in the NutriNet-Santé study

**Supplemental Tables and Figures**

**Supplemental Table 1. Construction of the SDI as computed by Seconda et al.** ^(1)^

| **Dimension** | **Indicator** | **Attribution of points for each indicator** | | | | | **Weighting used for each indicator** | **Overall scoring** |
| --- | --- | --- | --- | --- | --- | --- | --- | --- |
|  |  | **Q1** | **Q2** | **Q3** | **Q4** | **Q5** |  |  |
| **Nutrition** | dietary energy balance  PANDiet index | 5  1 | 4  2 | 3  3 | 2  4 | 1  5 | 0.5  0.5 | 5 |
|  |  |  |  |  |  |  |  |  |
| **Environment** | pReCiPe  proportion of organic food in the whole diet | 5  1 | 4  2 | 3  3 | 2  4 | 1  5 | 0.75  0.25 | 5 |
|  |  |  |  |  |  |  |  |  |
| **Sociocultural** | food supply source index  consumption frequency of ready-made foods | 1  5 | 2  4 | 3  3 | 4  2 | 5  1 | 0.5  0.5 | 5 |
|  |  |  |  |  |  |  |  |  |
| **Economic** | proportion of the income devoted to diet | 5 | 4 | 3 | 2 | 1 | 1 | 5 |

**Supplemental Figure 1. Study sample selection**

**37,685 participants completed the Org-FFQ**

37,305 participants had no missing covariates

34,453 participants were not living overseas

35,196 participants were not under/over-reporters

29,210 participants had place of purchase data

29,186 participants had available data for SDI computation

29,119 participants had available information on their current or last status occupation category

Final study sample: 29,119 participants

**Supplemental Figure 2. Average diet monetary cost (€/d) distinguishing the farming system of food consumed, according to mSDI quintiles (weighted data)**^1^


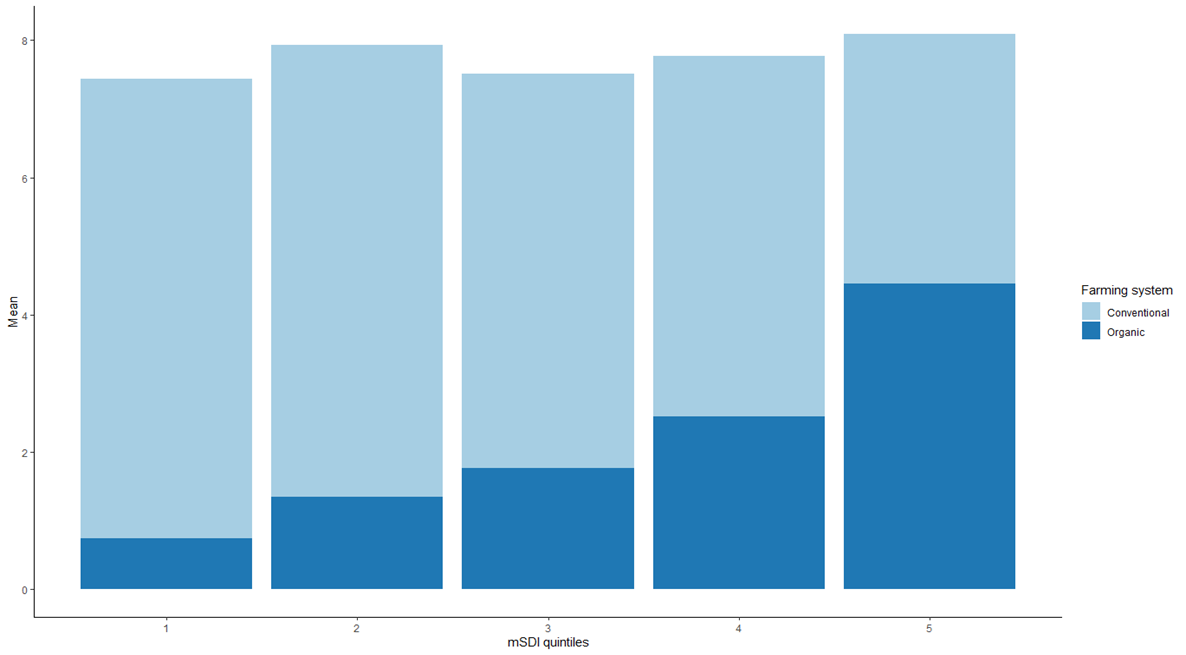


Abbreviation: mSDI, modified Sustainable Diet Index. P-value for linear contrast <.0001.

^1^Data are unadjusted.

**Supplemental Table 2: Association between socioeconomic factors and individual subscores, Poisson regression coefficients and 95%CI, according to sex (weighted data)^1^**

|  | Females | | | Males | | |
| --- | --- | --- | --- | --- | --- | --- |
|  | **Environment subscore** | **Sociocultural subscore** | **Nutritional subscore** | **Environment subscore** | **Sociocultural subscore** | **Nutritional subscore** |
| **Income per household unit, %** |  |  |  |  |  |  |
| <1200 €/month | 0.0210 (-0.014, 0.0564) | **-0.1005 (-0.136, -0.065)** | -0.0354 (-0.070, -0.000) | **0.1495 (0.1112, 0.1878)** | -0.0179 (-0.053, 0.0176) | **0.0523 (0.0131, 0.0915)** |
| 1200–1800 €/month | 0.0307 (-0.002, 0.0635) | **-0.0792 (-0.112, -0.047)** | -0.0115 (-0.044, 0.0208) | **0.0494 (0.0147, 0.0841)** | **-0.0846 (-0.117, -0.052)** | 0.0074 (-0.028, 0.0426) |
| 1800–2700 €/month | **0.0580 (0.0254, 0.0905)** | 0.0015 (-0.030, 0.0333) | 0.0016 (-0.031, 0.0337) | 0.0315 (-0.002, 0.0652) | -0.0034 (-0.034, 0.0274) | **-0.0721 (-0.107, -0.038)** |
| >2700 €/month | Ref | Ref | Ref | Ref | Ref | Ref |
| Refuse to declare | 0.0108 (-0.031, 0.0526) | **-0.0597 (-0.101, -0.018)** | -0.0067 (-0.048, 0.0344) | **-0.0909 (-0.149, -0.033)** | **-0.0666 (-0.120, -0.014)** | -0.0319 (-0.091, 0.0266) |
| **Educational level, %** |  |  |  |  |  |  |
| Primary | 0.0092 (-0.028, 0.0467) | **-0.0517 (-0.090, -0.014)** | 0.0015 (-0.036, 0.0394) | **-0.1292 (-0.174, -0.085)** | **-0.1078 (-0.149, -0.066)** | **-0.1767 (-0.223, -0.131)** |
| Secondary | **-0.0391 (-0.073, -0.005)** | **-0.0647 (-0.099, -0.030)** | -0.0123 (-0.047, 0.0222) | **-0.0810 (-0.119, -0.043)** | **-0.0844 (-0.120, -0.049)** | **-0.1126 (-0.152, -0.073)** |
| Undergraduate | **-0.0534 (-0.091, -0.016)** | **-0.0421 (-0.080, -0.004)** | 0.0102 (-0.028, 0.0482) | 0.0078 (-0.038, 0.0530) | -0.0366 (-0.079, 0.0059) | **-0.0598 (-0.107, -0.012)** |
| Postgraduate | Ref | Ref | Ref | Ref | Ref | Ref |
| **Occupational status, %** |  |  |  |  |  |  |
| Self-employed (e.g. farmer/entrepreneur) | 0.0128 (-0.033, 0.0590) | **0.0810 (0.0362, 0.1258)** | **0.0567 (0.0106, 0.1028)** | 0.0022 (-0.040, 0.0442) | **0.1032 (0.0648, 0.1416)** | **-0.1058 (-0.151, -0.061)** |
| Never-employed (e.g. student) | **-0.0520 (-0.088, -0.016)** | **-0.0747 (-0.111, -0.038)** | -0.0027 (-0.039, 0.0335) | 0.0022 (-0.040, 0.0442) | 0.0198 (-0.030, 0.0691) | **0.1096 (0.0563, 0.1630)** |
| Manual worker | **-0.1268 (-0.172, -0.082)** | **-0.0454 (-0.089, -0.002)** | -0.0066 (-0.050, 0.0371) | **-0.0962 (-0.130, -0.062)** | **-0.0576 (-0.090, -0.026)** | -0.0346 (-0.070, 0.0003) |
| Employee/blue collar | **-0.0651 (-0.091, -0.039)** | **-0.1200 (-0.146, -0.094)** | -0.0156 (-0.042, 0.0102) | **-0.0766 (-0.114, -0.039)** | **-0.0727 (-0.109, -0.037)** | **-0.0578 (-0.097, -0.019)** |
| Intermediate profession | Ref | Ref | Ref | Ref | Ref | Ref |
| Managerial staff/intellectual profession | **-0.0382 (-0.074, -0.003)** | -0.0333 (-0.068, 0.0017) | **0.0386 (0.0036, 0.0737)** | -0.0069 (-0.042, 0.0278) | 0.0145 (-0.018, 0.0470) | -0.0057 (-0.041, 0.0299) |

Bold denotes significance.

^1^Multivariable Poisson regression for the association between the individual component and each socio-economic factor. Multivariable-adjusted models include age, total energy intake, parental status, residential area, and socio-economic factors. The socio-economic factors are mutually adjusted for the remaining factors.

**Supplemental material**

*Assessment of dietary intakes*

Nutrient intakes independently of the food production method (due to lack of data for organic food) were calculated using a food composition database developed within the NutriNet-Santé study ^(2)^. Schofield equations, which accounts for basal metabolic rate and physical activity levels, were used to estimate energy requirement ^(3)^.

To estimate the nutritional quality of the diet, we computed the PANDiet (Diet Quality Index Based on the Probability of Adequate Nutrient Intake), an index which measures the probability of adequacy to the French nutritional references for 27 nutrients and of moderation for 6 nutrients. The PANDiet also penalizes excessive intakes of 12 nutrients. The PANDiet ranges from 0 to 100, higher values reflecting higher compliance with the French national reference values ^(4)^. Full details on the PANDiet calculation can be found elsewhere ^(5,6)^.

*Assessment of environmental pressures and impact*

Environmental pressures, which included GHGe, land use and cumulative energy demand, were primarily derived from the comprehensive French tool DIALECTE ^(7)^, using an LCA approach at the farm level, completed by existing literature (8). Environmental pressures in organic and conventional were estimated for 92 raw products (components of 442 ingredients composing the 264 Org-FFQ items). Economic allocations and edible coefficients were then applied to estimate the impact of the food as consumed. Food production-related pressures at the individual level were obtained by merging consumption data with environmental data, while considering the food production method (organic or conventional). The system boundaries were cradle-to-farm gate, meaning that transport-, storage- or loss-related impacts were excluded from the evaluation. More information on the environmental assessment can be found elsewhere ^(8,9)^. The pReCiPe (partial ReCiPe), an overall impact score which enables to consider possible conflicts between different environmental footprints, was computed. It comprises three indicators (greenhouse gas emissions (in kg CO2_eq_), primary energy consumption (in MJ) and land occupation (in m2)), weighted by means of coefficients ^(1,10)^. The formula used to compute the pReCiPe is the following: pReCiPe = 0.0459 × greenhouse gas emissions (in kg CO_2eq_/kg) + 0.0025 × primary energy consumption (in MJ/kg) + 0.0439 × land occupation (in m^2^/kg). We calculated individual’s diet pReCiPe by multiplying the pReCiPe of each item by its quantity consumed daily by the individual (while considering the food production method) and then summing up the total. A higher pReCiPe indicates a higher environmental impact.

*Assessment of food purchase place and eating practices*

Data related to ready-made food consumption as a proxy for eating practices and food places of purchase were collected via a dedicated self-administered questionnaire that was proposed in the same period as the Org-FFQ. The questionnaire is described more in depth elsewhere ^(9,11)^.

More specifically, participants completed information of their main food source supply for ca. 30 (organic and conventional) food categories. An index was computed to estimate sustainability food purchase places. Two points were allocated to the following sources of supply: producers’ markets, farmer shops, baskets from AMAP (French associations supporting small-scale local producers), craft stores, farms or self-production, one point to markets, groceries, specialised organic shops or cooperatives, and zero to supermarkets. Then, the index was the sum of the points divided by the total number of food purchase places cited by the participant.

Additionally, an individual’s index expressing consumption of 3 types of ready-made dishes (i.e. canned, frozen and ready-made foods) was calculated by attributing weights (0, 0.25, 0,5, 0.75, 1) to the respective frequency modality (never, rarely, half of the time, often, and always) and summing up the total.

*Assessment of monetary diet cost*

For each place of purchase, an average price was assigned to each item of the Org-FFQ in organic and conventional.

To do so, a specially price database was developed. In brief, price data were primarily derived from the 2012 Kantar Worldpanel purchase database ^(12)^ for products purchased from supermarkets and certain specialty stores, completed by additional prices collected by Bioconsom’acteurs’ association for short supply channels (e.g. local markets). Thus, 1100 prices were collected by Bioconsom’acteurs members in the fall of 2014 and 862 in the spring of 2015 across nine departments of different French regions, using a standardised procedure. The Kantar dataset is a home-scan dataset which provides detailed information on all food product purchase of a representative consumer panel of 20,000 French households.

We assessed the individual daily diet monetary cost, i.e. the monetary value of the diet in € per day, by combining the amounts of each food consumed (g/d) by the related item price (€/g), accounting for cooking preparation, the method of food production, and the source of supply (assessed by food categories, as explained above).

**Supplemental discussion**

*Associations by sex*

In the present study, findings regarding higher educational level and a more sustainable diet did not considerably vary according to sex, although the associations appeared strong in males. The scientific literature about differential associations by sex between education and diet quality has led to inconsistent findings. For instance, in a study conducted in Australia, the relationship between diet quality assessed using the Dietary Guideline Index and education level was slightly stronger among females ^(13)^ while in a study conducted in Denmark using the Healthy Eating Index the association was significant in males but not in females.

We observed opposite results for sex within low-income individuals (<1200€/month). Low-income females had lower diet sustainability than their most privileged counterparts in contrast to low-income males who had higher diet sustainability, despite adjustment for age and occupational status.

The same trend was observed among never-employed individuals. Although models were adjusted for age, one possible explanation could be different subcategory distributions within this group depending on sex. Never-employed were mostly homemakers in females while they were mostly students in males, re-emphasising that education and associated health literacy seem important drivers for sustainable eating habits.

**References cited in the present document**

1. Seconda L, Baudry J, Pointereau P, et al. (2019) Development and validation of an individual sustainable diet index in the NutriNet-Santé study cohort. *Br. J. Nutr.* **121**, 1166–1177.

2. Etude Nutrinet-Santé (2013) Etude Nutrinet-Santé. Table de composition des aliments de l’étude Nutrinet-Santé (Nutrinet-Santé Study Food Composition Database). Paris: Economica. .

3. Schofield WN (1985) Predicting basal metabolic rate, new standards and review of previous work. *Hum Nutr Clin Nutr* **39 Suppl 1**, 5–41.

4. French Agency for Food & Safety (ANSES) OH& (2016) Actualisation des repères du PNNS: élaboration des références nutritionnelles. Anses Maisons-Alfort (France).

5. Verger EO, Mariotti F, Holmes BA, et al. (2012) Evaluation of a Diet Quality Index Based on the Probability of Adequate Nutrient Intake (PANDiet) Using National French and US Dietary Surveys. *PLoS ONE* **7**, e42155.

6. De Gavelle E, Huneau J-F & Mariotti F (2018) Patterns of Protein Food Intake Are Associated with Nutrient Adequacy in the General French Adult Population. *Nutrients* **10**, 226. Multidisciplinary Digital Publishing Institute.

7. Pointereau P, Langevin B, Gimaret M, et al. (2012) DIALECTE, a comprehensive and quick tool to assess the agro-environmental performance of farms. In *10th European IFSA Symposium, July*, pp. 1–4.

8. Seconda L, Baudry J, Allès B, et al. (2018) Comparing nutritional, economic, and environmental performances of diets according to their levels of greenhouse gas emissions. *Climatic Change* **148**.

9. Kesse-Guyot E, Lairon D, Allès B, et al. (2021) Key Findings of the French BioNutriNet Project on Organic Food–Based Diets: Description, Determinants, and Relationships to Health and the Environment. *Advances in Nutrition*.

10. Kramer GF, Tyszler M, Veer PV, et al. (2017) Decreasing the overall environmental impact of the Dutch diet: how to find healthy and sustainable diets with limited changes. *Public Health Nutr* **20**, 1699–1709.

11. Baudry J, Pointereau P, Seconda L, et al. (2019) Improvement of diet sustainability with increased level of organic food in the diet: findings from the BioNutriNet cohort. *Am. J. Clin. Nutr.* **109**, 1173–1188.

12. Kantar Worldpanel Consumer Panels. https://www.kantarworldpanel.com/global (accessed July 2017).

13. Backholer K, Spencer E, Gearon E, et al. (2016) The association between socio-economic position and diet quality in Australian adults. *Public Health Nutrition* **19**, 477–485. Cambridge University Press.
